# Tweaking the Structure to Radically Change the Function: The Evolution of Transthyretin from 5-Hydroxyisourate Hydrolase to Triiodothyronine Distributor to Thyroxine Distributor

**DOI:** 10.3389/fendo.2014.00245

**Published:** 2015-02-11

**Authors:** Samantha J. Richardson

**Affiliations:** ^1^School of Medical Sciences, RMIT University, Bundoora, VIC, Australia

**Keywords:** 5-hydroxyisourate hydrolase, mechanism, prealbumin, protein evolution, transthyretin, transthyretin-like protein, triiodothyronine, thyroxine

## Abstract

Often, we elucidate evolutionary processes backwards, starting with eutherian mammals and gradually climbing down the evolutionary tree to those species who have survived since long before mammals evolved. This is also true for elucidating the evolution of specific proteins, in this case, the protein currently known as “transthyretin” (TTR). TTR was first described in eutherian mammals and was known as a thyroxine (T4) binding protein. However, mammals are the exception among vertebrates in respect to the function of TTR, as in teleost fish, amphibians, reptiles and birds TTR preferentially binds triiodothyronine (T3), which is the active form of thyroid hormone (TH). The TTR gene possibly arose as a duplication of the transthyretin-like protein (TLP) gene, around the stage of the agnathans. Some vertebrate species have both the TTR and TLP genes, while others have “lost” the TLP gene. TLP genes have been found in all kingdoms. The TLPs analyzed to date do not bind THs or their analogs, but are enzymes involved in uric acid metabolism; specifically, they are 5-hydroxyisourate hydrolases. A *Salmonella* TLP knock-out strain demonstrated that TLP was essential for the bacteria’s survival in the high uric acid environment of the chicken alimentary tract. Many other TLPs are yet to be characterized for their function although several have been confirmed as 5-hydroxyisourate hydrolases. This review describes the evolution of TLP/TTR and how subtle changes in gene structure or amino acid substitution can drastically change the function of this protein, without altering its overall 3D conformation.

## The Role of Transthyretin in Thyroid Hormone Distribution

**Figure 1:**
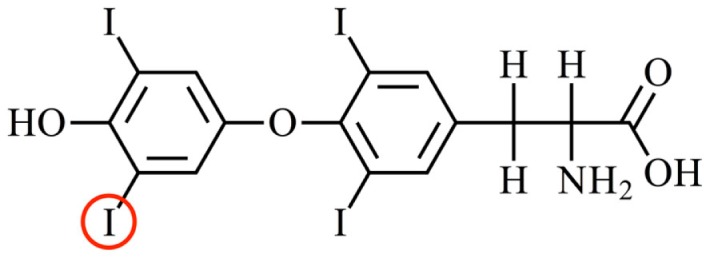
**The structure of the main thyroid hormone thyroxine**. Thyroxine (3′,5′,3,5-tetraiodo-l-thyronone; T4) is the predominant form of thyroid hormone secreted by the thyroid gland into the blood. T4 is converted to T3 (5′,3,5,-triiodo-l-thyronine) by an enzymatic removal of an iodine atom from the outer ring of T4. For example, the iodine atom circled in red. Two enzymes that perform this function are deiodinase 1 (e.g., in the liver) and deiodinase 2 (e.g., in the brain). T3 is the predominant form of thyroid hormone that binds to the nuclear thyroid hormone receptors.

Thyroid hormones (THs) are involved in the regulation of growth, development and metabolism. There are two main forms of THs: 3′,5′,3,5-tetraiodo-l-thyronine (thyroxine, T4) and 5′,3,5-triiodo-l-thyronine (T3) (Figure 1). The only site of TH synthesis is the thyroid gland, which secretes the THs (predominantly as T4) into the blood. THs are lipophilic and preferably partition into the lipid phase rather than the aqueous phase (1). To prevent the avid partitioning of THs into the membranes of the first cells they encounter, there are specific proteins in the blood that bind and distribute THs, thereby creating a circulating pool of sufficient size to distribute THs from their site of synthesis (the thyroid gland) via the aqueous environment of the blood stream to their sites of action, i.e., cells throughout the body (2, 3). In humans, there are three TH distributor proteins in the blood: albumin, transthyretin (TTR), and thyroxine-binding globulin. Of these three proteins, albumin is present in highest abundance but binds THs with lowest affinity, thyroxine-binding globulin is present in lowest abundance but binds THs with highest affinity, and TTR is present in intermediate abundance and binds with intermediate affinity. Taking into consideration the affinities these proteins have for THs and the capillary transit times through tissues, albumin binds so weakly that the amount of TH that it delivers is extremely low; thyroxine-binding globulin binds THs so tightly, that the amount of TH it delivers is also low; whereas TTR binds THs with an intermediate affinity rendering it the most significant in terms of TH delivery to tissues [for a detailed quantitative analysis, see Richardson (4)]. This can be seen as analogous to the situation for Goldilocks and the Three Bears.

**Figure 2:**
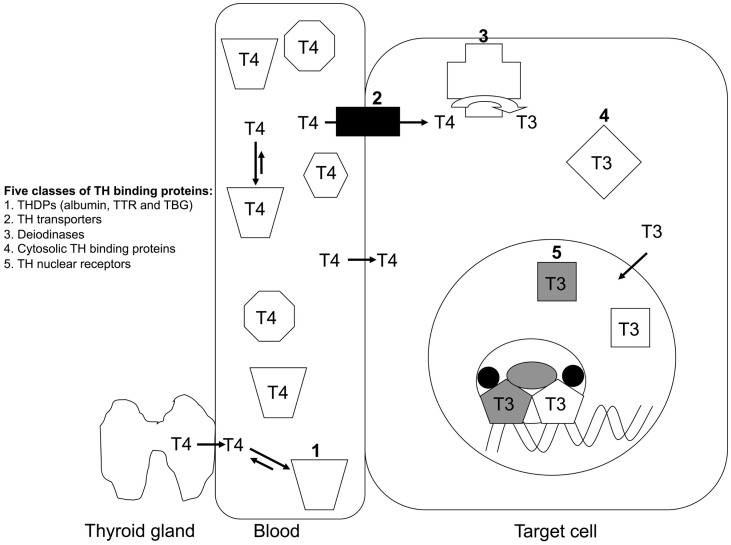
**The five classes of TH-binding proteins**. T4 is the predominant form of TH secreted by the thyroid gland in mammals. T4 is the predominant form of TH in mammalian blood and T3 is also present in significant quantities, but for simplicity T4 is the only form of TH shown in blood in this schematic. More than 99% of TH (both T4 and T3) in blood is bound to a TH distributor protein (1) e.g. albumin, transthyretin (TTR) or thyroxine-binding globulin. TH can dissociate from its distributor protein and enter cells via either TH transporters (2) or by diffusion. Inside the cell, THs can be deiodinated by a family of deiodinases (3), which can either activate (e.g. T4 to T3) or inactive (e.g. T3 to T2) the TH. THs can bind to cytosolic proteins (4). Predominantly T3 binds to TH nuclear receptors (5), which bind to specific regulatory regions of TH-regulated genes and protein complexes to either promote or repress transcription of those genes (4).

Once THs are bound to a TH distributor protein, with the affinity determined by the on and off rates, the TH can dissociate and enter a cell, either by diffusion (1) or via a membrane-bound TH transporter (5). Inside the cell, the TH can then be activated or inactivated by a family of enzymes called deiodinases (6). T4 is known as the “transport form” of TH, as it is the predominant form present in blood, whereas T3 is known as the “active form” of TH as it has higher affinity for the TH receptors (7), which are nuclear transcription factors. Thus, deiodinases can activate T4 to T3, or can inactivate T4 to reverse T3 (rT3) or inactivate T3 to T2 (6). T3 can bind to cytosolic proteins and also to the thyroid hormone receptors (TRs), which can translocate into the nucleus, dimerize, recruit co-modulator proteins, and regulate transcription of specific genes. Many such genes are involved in growth, development, and metabolism, a spectacular example being metamorphosis of a tadpole (aquatic, herbivorous, gills, tail for locomotion) to a frog (terrestrial, carnivorous, lungs, four limbs for locomotion) [see Shi (8)]. Thus, TTR is a member of one of the five known classes of TH-binding proteins (TH distributor proteins; TH transporter proteins; deiodinases; cytosolic proteins; nuclear receptors) (Figure 2).

**Figure 3:**
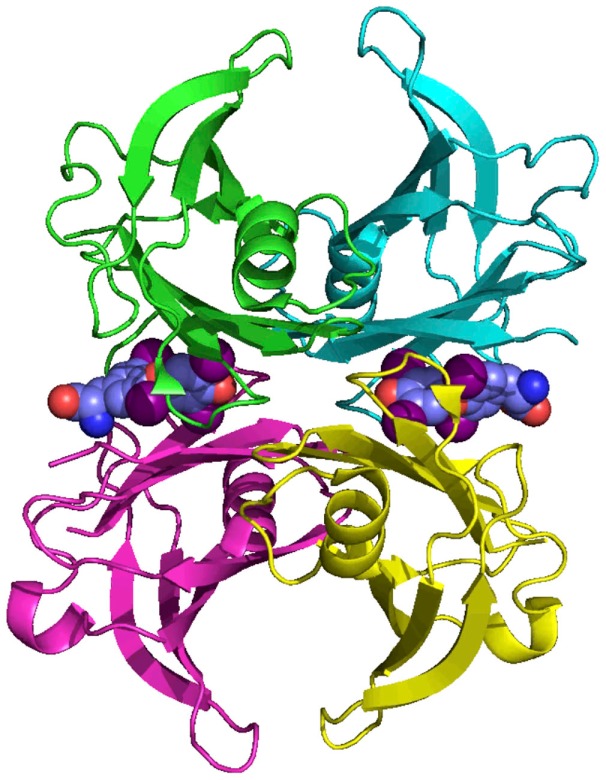
**The structure of human TTR**. TTR is a homo-tetramer with a central channel that contains two potential thyroid hormone binding sites. Each subunit is rich in beta-sheet structure. Coordinates from Blake et al. (9).

Transthyretin is a homo-tetramer, held together by non-covalent interactions, without post-translational modifications. Each subunit comprises eight β-strands that form two β-sheets and a short region of α-helix (Figure 3). The holo-protein has a central channel, which has two TH-binding sites (9); however, under physiological conditions only one site is occupied, due to negative co-operativity (10).

## TTR Null Mice Have a Subtly Altered Phenotype, But Humans Lacking TTR Have Not Been Described

Mice in which the TTR gene has been inactivated [TTR null mice; (11)] have delayed development of several TH-regulated events including central nervous system development, growth of long bones, suckling-to-weaning transition (12), and adult TTR null mice have a hypothyroid phenotype in the central nervous system (13). Thus, the role of TTR in TH distribution in eutherians has been demonstrated, despite having additional TH distributor proteins (albumin, TTR and thyroxine-binding globulin in the blood are all synthesized by the liver and secreted into the blood). Humans lacking TTR have not been documented, although humans lacking albumin and thyroxine-binding globulin have been reported [see Harms et al. (14)]. Possibly, this is because TTR is the only TH distributor protein synthesized in the central nervous system: in the choroid plexus, which forms the blood–cerebrospinal fluid barrier. This TTR has been implicated in moving TH from the blood into the cerebrospinal fluid (1, 15, 16).

## Shortening of the N-Terminal Region of TTR Resulted in Changing the Ligand from T3 to T4

**Figure 4:**
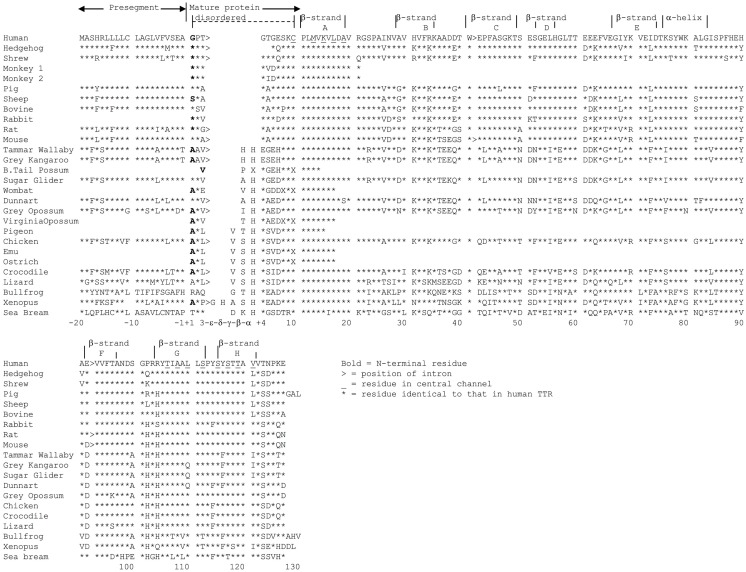
**Alignment of representative TTRs from various vertebrate species**. Amino acid sequences (and those derived from cDNA sequences) of TTRs from species including eutherians, marsupials, birds, reptiles amphibians, and a fish were aligned. Asterisks indicate that amino acid is identical to that in human TTR. Gaps were introduced to aid alignment. Residues in bold are the N-terminal amino acid. Positions of introns are indicated by >. Residues in the central channel are underlined (4).

**Figure 5:**
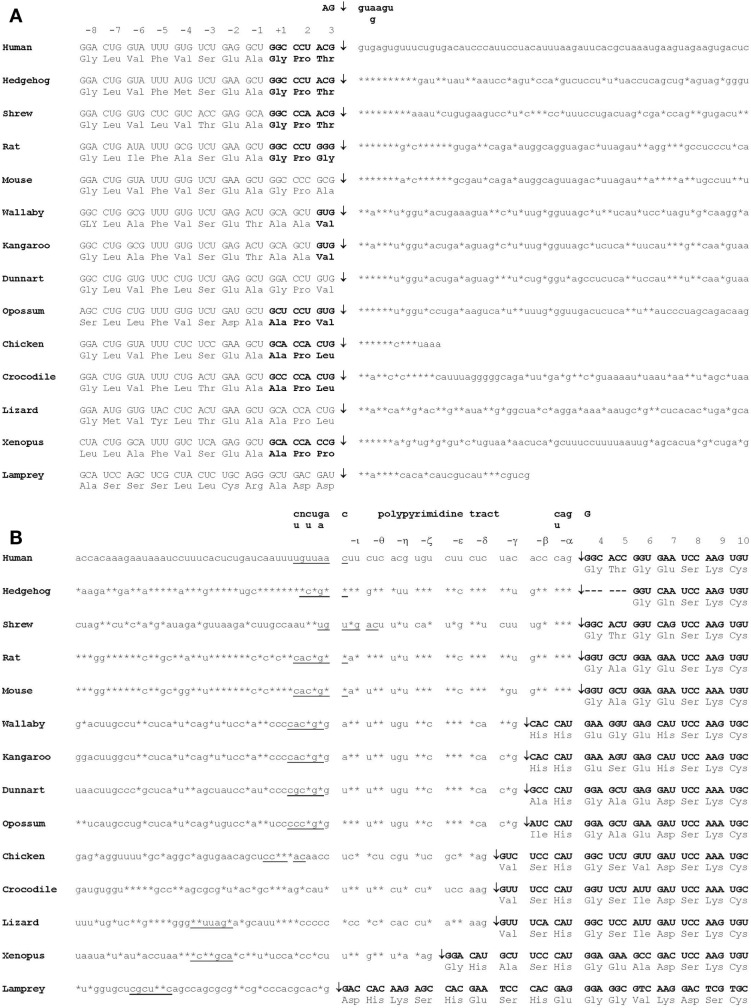
**Mechanism of shortening of the N-terminal region of TTR during vertebrate evolution**. **(A)** The position of the exon 1–intron 1 splice site of the TTR precursor mRNA did not change during vertebrate evolution. **(B)** The position of the intron 1–exon 2 splice site of the TTR precursor mRNA shifted in the 3′ direction during vertebrate evolution. Double-headed arrows indicate the positions of splice sites. Consensus splicing site sequences are underlined. Asterisks indicate an identical base to that in human TTR precursor mRNA. N-terminal regions are boxed (19).

The amino acid sequence has been determined or derived from cDNA sequences for TTRs from more than 20 vertebrate species including teleost fish, amphibians, reptiles, birds, and mammals. The amino acid sequence has been highly conserved throughout vertebrate evolution, in particular, the amino acids which correspond to regions involved in monomer–monomer interactions, dimer–dimer interactions and those involved in TH binding [see Prapunpoj et al. (17)]. The region of TTR which has changed the most during vertebrate evolution is the N-terminal region, which has changed from longer (e.g. in amphibians) to shorter (e.g. in eutherian mammals). This has occurred in a step-wise manner, which implies a specific and persistent selection pressure acting on TTR during vertebrate evolution (Figure 4). Comparison of the cDNA with the genomic DNA in this region for each species revealed that the exon 1–exon 2 border was in the region of the gene corresponding to the N-terminal region of the protein subunit. Further analyses revealed that the position of the exon 1–intron 1 border did not change during vertebrate evolution. However, the position of the intron 1–exon 2 border appears to have shifted in the 3′ direction, in a step-wise manner, due to a series of single base changes in the gene. Thus, the mechanism for the shortening of the N-terminal regions of TTRs can be explained by a series of changes in the gene that “moved” an increasing number of bases from exon 2 into intron 1 (18) (Figure 5).

The effect of moving a series of bases of the TTR gene from exon 2 to intron 1 shortened the N-termini of the TTR tetramer but, most importantly, changed the function of TTR. Perhaps, this was the driving selection pressure for the movement of the intron 1–exon 2 splice site in the 3′ direction.

Transthyretins with longer N-terminal regions (including those from teleost fish, amphibians, reptiles and birds) have higher affinity for T3 than for T4 (17, 19–22), whereas TTRs with shorter N-terminal regions (including those from marsupials and eutherians) have higher affinity for T4 (21) (Table 1). Thus, mammalian TTRs are the exception binding T4 > T3, as TTRs from all other classes of vertebrates bind T3 > T4. The hypothesis that binding of T3 or T4 was due to the N-terminal regions of TTRs was tested by two separate studies. The first study involved the purification of TTR from chicken blood followed by elucidation of the X-ray crystal structure of chicken TTR, to determine if there were structural changes in the TH-binding site between human TTR (which preferentially binds T4) and a TTR that preferentially bound T3. The structure of the TH-binding site in chicken TTR did not differ to that of human TTR (23). Thus, a different region of the molecule must be responsible and the best candidates were the N-terminal regions, which move freely in solution around the entrances to the channel containing the TH-binding sites. The second study involved a set of recombinant TTRs, including chimeric TTRs whose N-terminal regions had been swapped (e.g. N-terminal region of crocodile TTR attached to the “body” of human TTR and vice versa). These TTRs were analyzed for their affinities to T3 and T4. Indeed, the structure of the N-terminal regions did influence the affinity and preference of ligand binding (24).

**Table 1:** **Comparison of affinities of TTRs for T4 and T3 (4)**.

Source of TTR	Kd T4 (nM)	Kd T3 (nM)	Kd T3/Kd T4
Eutherians
Human	13.6	56.6	4.2
Sheep	11.3	63.5	3.2
Rat	8.0	67.2	8.4
Marsupials
Wombat	21.8	97.8	4.5
Possum	15.9	206.1	12.9
Wallaby	13.8	65.3	4.7
Birds
Emu	37.4	18.9	0.51
Chicken	28.8	12.3	0.43
Pigeon	25.3	16.1	0.64
Reptile
Crocodile	36.7	7.56	0.21
Amphibian
Toad	508.0	248.0	0.49

Thus, by shifting the position of the intron 1–exon 2 border, TTR was able to change from being a T3 distributor to a T4 distributor. What could the selection pressure have been for changing the ligand of TTR from T3 to T4? T3 is the active form of the hormone whereas T4 is the pro-hormone. Perhaps, distributing the pro-hormone could be considered safer than distributing the active form of the hormone, requiring an additional level of activation of the pro-hormone by tissue-specific deiodinases, which are very tightly regulated in terms of developmental and tissue-specificity. In particular, this could be important in the central nervous system, as in (for example) the rat brain, the proportion of T3 generated by local deiodination of T4 is specific to the region e.g. 65% in the cortex, 51% in the cerebellum, 35% in the pons, 32% in the hypothalamus, 30% in the medulla oblongata and 22% in the spinal cord (25). Such tight regulation might not be possible if T3 were the predominant form of TH in the blood and cerebrospinal fluid.

To put this into context, we should consider the total T4 and total T3 levels in blood from various classes of vertebrates. A comprehensive review by Hulbert (26) has tabulated the concentrations of T3 and T4 in the blood of more than 80 vertebrate species (including several life stages for several species). In mammals, birds and reptiles, the circulating levels of T4 are higher than those of T3. However, in some amphibians and teleost fish, the levels for circulating T3 and T4 are similar, while in other species the levels of T4 are higher than those of T3. Of particular interest, those animals undergoing metamorphosis or smolting have the characteristic peak in TH concentrations in their blood. In some species, this has been correlated with a transient expression of TTR (teleost fish; amphibian; reptile; polyprotodont marsupial) or TBG (diprotodont marsupial) (27). Speculation as to the driving force for greater control of T3 availability in specific brain areas in mammals, as opposed to other vertebrates is very tempting, but extremely speculative. Key TH-related features that distinguish mammalian brains from those of other vertebrates include (i) the corpus callosum and its extensive myelination and (ii) the highly developed cerebral cortex. Furthermore, mammalian fetuses have low levels of circulating T3 and maternal T4 (not T3) has a crucial role in brain development (28).

## The TTR Gene Arose as a Duplication of the TLP Gene

**Figure 6:**
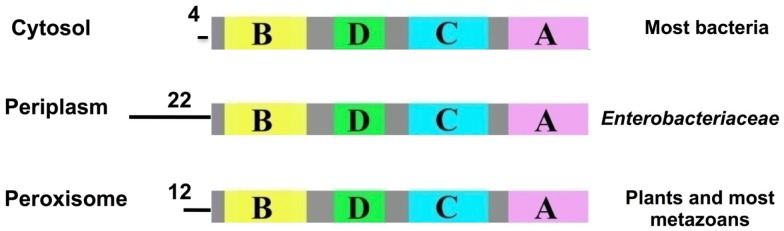
**Predicted signal peptides of TLPs**. The signal-peptide prediction predicted three main groups for TLPs: cytosolic (most bacteria), periplasmic (enterobacteria) and peroxisomal (plants and most metazoans). Data from Hennebry et al. (29).

As mentioned earlier, the amino acid sequence of TTR has been highly conserved during vertebrate evolution, to the extent that the gene most likely arose prior to the divergence of vertebrates from non-vertebrates. Therefore, open reading frames which would code for transthyretin-like proteins (TLPs) were searched for and identified (17). Subsequently, as increasing numbers of genomes were sequenced, genes coding for more than 80 potential TLPs have been identified (and verified to be full-length and not to contain in-frame stop codons, etc.) in all kingdoms (29). Phylogenetic analysis revealed that TLP sequences generally clustered according to organism groups. Vertebrate TLPs clustered together, close to TTRs. TTRs were only found in vertebrate species, whereas TLP sequences were found in both vertebrate and non-vertebrate species (29). Analyses of the TLP gene sequences by signal-peptide prediction programs revealed that TLPs could be divided into three groups: (1) those without signal peptides were predicted to be cytoplasmic, which included most bacterial TLPs; (2) those with periplasmic localization signals, which were the enterobacteria; (3) those with peroxisomal (PTS2) signal peptides, which included TLPs from plants and most metazoans (29) (Figure 6). Several bacterial species have more than one copy of the TLP gene, often one copy that codes for a cytoplasmic TLP and another copy coding for a periplasmic TLP (29). A neighbor-joining tree analyzing the relationship between cytoplasmic and periplasmic TLPs showed that within a given species, cytoplasmic TLPs clustered separately to periplasmic TLPs. For example, all periplasmic TLPs group together. This suggested that periplasmic TLP sequences probably evolved along a separate evolutionary pathway to cytoplasmic TLPs (30). Thus, TLPs appear to have evolved different functions, depending on their sub-cellular localization, rendering them very versatile proteins. These characteristics of TLPs which result from the variety of sub-cellular localizations contrast with TTRs, which are secreted.

## Subtle Changes in the Active Site Changed TLPs (Enzymes Involved in Uric Acid Oxidation) into TTRs (Thyroid Hormone Distributors)

**Figure 7:**
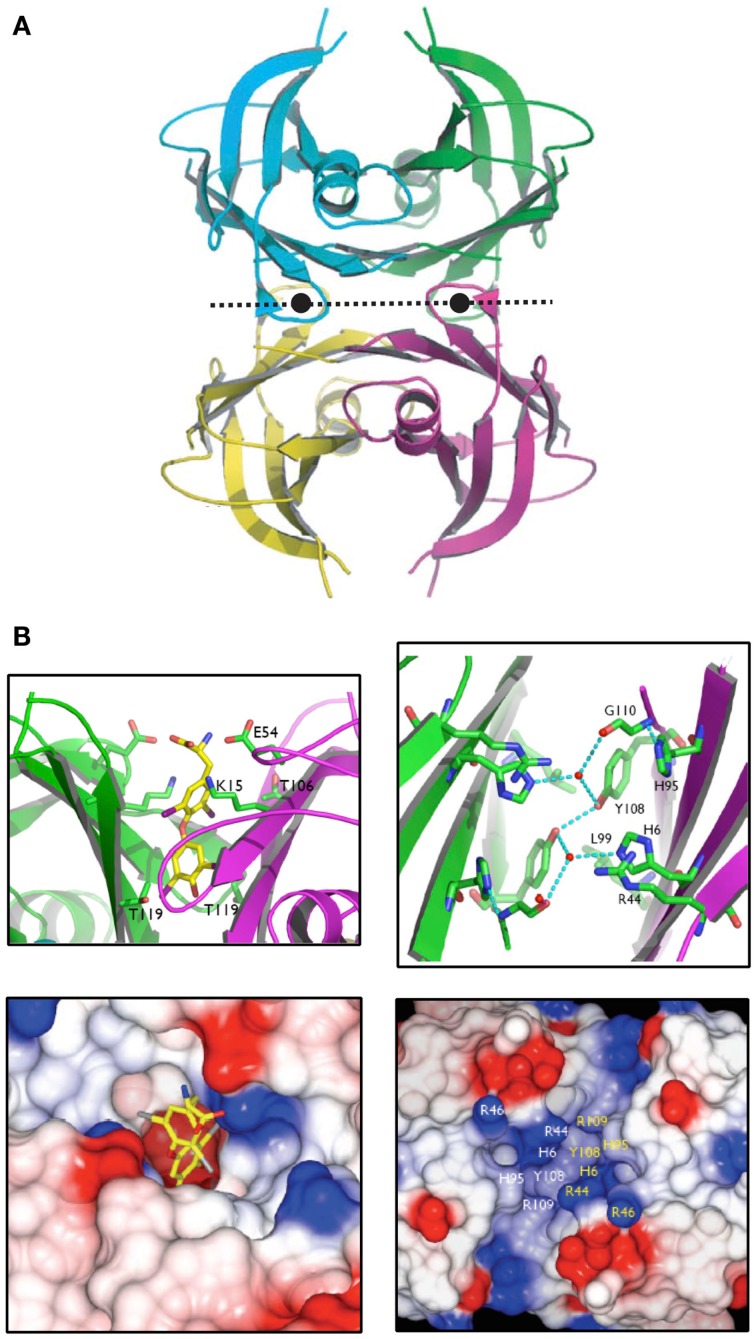
**The structure of *Salmonella dublin* TLP**. **(A)**. The X-ray crystal structure of *S. dublin* TLP was that first TLP structure to be determined. The overall structure is almost identical to that of human TTR. **(B)**. The thyroid hormone binding site in human TTR is deep and negatively charged, whereas the equivalent site in *S. dublin* TLP is shallow and positively charged (31).

**Figure 8:**
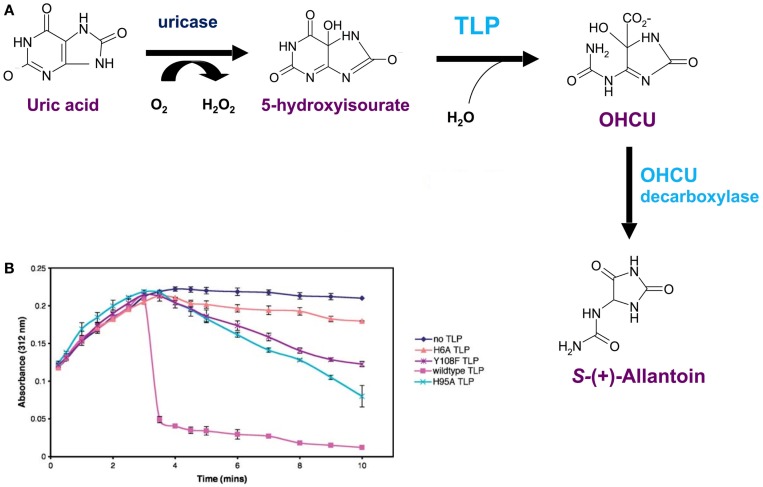
***Salmonella dublin* TLP is a 5-hydroxyisourate hydrolase**. **(A)** The uricase pathway, responsible for the oxidation of uric acid to allantoin. **(B)** Hydrolysis of 5-hydroxyisoirate (5-HIU) by *S. dublin* TLP and three mutated forms: H6A, H95A and Y108F. Uric acid was added to the solution containing uricase and 5-HIU was monitored at 312 nm. Maximum 5-HIU was generated after 3 min and then spontaneously decomposed (black line). Addition of *S. dublin* TLP resulted in rapid hydrolysis of 5-HIU (magenta line). Rapid hydrolysis of 5-HIU did not occur for the mutants H6A, H95A and Y108F (31).

It was revealed by PCR that the TLP genes from a plant (*A. thaliana*), a worm (*C. elegans*) and bacteria (*E. coli* and *S. dublin*) were expressed in their respective species i.e. these open reading frames were genes that were expressed in nature. The respective cDNAs were cloned, sequenced and recombinant TLPs from these species were synthesized and found to be tetramers, similarly to TTR. However, these TLPs did not bind THs or TH analogs (29). The X-ray crystal structure of recombinant *Salmonella dublin* TLP was determined (Figure 7A) and was completely superimposable over those of vertebrate TTRs (31). The only differences were subtle changes in the region equivalent to the TH-binding site. Whereas in TTRs the binding site is deep and negatively charged, the equivalent position in TLP was shallow, elongated and positively charged, thereby preventing binding of THs (Figure 7B). Careful analysis of the operons within which several TLP genes were situated, allowed the identification of TLP in (at least several) bacteria as a 5-hydroxyisourate hydrolase (5-HIUase), involved in the oxidation of uric acid to allantoin (Figure 8A). This was confirmed experimentally for *S. dublin* TLP, including identification of some of the amino acids required for catalysis (Figure 8B) (31) and has also been confirmed for TLP in *Bacillus subtilis* (32), zebra fish (33), mouse (34), *E. coli* (35), *Arabidopsis thaliana* (36), *Klebsiella pneumonia* (37), Coelacanth (38), amphioxus (39) and rainbow trout (40).

An insightful paper by Cendron and colleagues (41) identified two amino acid substitutions that were most probably fundamentally critical for the modification of the TLP active site ablating enzymatic activity and allowing opening up of the central channel to allow binding of THs. These are Ile to Ala at position 16 of zebrafish TLP (corresponding to position 19 of human TTR) and Tyr to Thr at position 116 of zebrafish TLP (corresponding to position 119 of human TTR). Li and colleagues demonstrated that in amphioxus (protochordate, close relative of vertebrates) TLP, the point mutation Tyr to Thr at position 156 (corresponding to position 116 in human TTR) was required for abolishing 5-HIUase activity and enabling T4 binding (39). While these mutations were probably instrumental in changing the landscape of the binding site from a shallow catalytic site to a deep channel, further minor mutations were probably required to optimize the channel for tighter binding of T3.

Interestingly, there are three splice variants of *Arabidopsis thaliana* TLP: two are cytoplasmic and one is located in the peroxisome (36). The peroxisomal isoform has a 5-HIUase domain and an OHCU decarboxylase domain i.e. both enzymes occur in the one transcript resulting in a bi-functional enzyme. There is an internal peroxisomal signal peptide between the two domains (N-terminal to the OHCU decarboxylase domain), presumably targeting the bi-functional TLP to the peroxisome, where uric acid degradation occurs. The function(s) of the cytoplasmic TLPs are not yet known. Furthermore, teleost fish, whose genomes have undergone an additional whole genome duplication, have two forms of 5-HIUase. One form contains the peroxisomal signal peptide and the other does not (40). Thus, in organisms where there have been further TLP gene duplications, such as *Arabidopsis* and (at least some) teleost fish, there is further scope for neo-functionalization of the TLP gene products i.e. the protein resulting from the duplicated TLP gene could acquire a different function by acceptance of point mutations in the duplicated gene while the original TLP gene/protein remains unchanged.

## In *Salmonella*, TLP is Required for Survival in High Uric Acid Environments

To demonstrate the function of *Salmonella* TLP in an animal model, a *Salmonella typhimurium* TLP knock-out strain was generated and its survival was compared with that of wildtype *Salmonella typhimurium*. The absence of TLP did not affect *Salmonella* survival in mice, whether the *Salmonella* were injected into the tail vein (and monitored for weight loss, development of enteric fever and bacterial load in liver or spleen) or infected orally (and bacterial load determined in Peyer’s Patches, mesenteric lymph nodes, liver and spleen) (30). Consequently, it was reasoned that if TLP was a 5-HIUase located in the periplasm of the *Salmonella*, then it would be important for the survival of the *Salmonella* in high uric acid environments such as the gastrointestinal tract of birds and reptiles (uric acid is present in high amounts in feces of reptiles and birds). Thus, mice might have been an inappropriate model animal for testing the effect of TLP on *Salmonella* survival.

**Figure 9:**
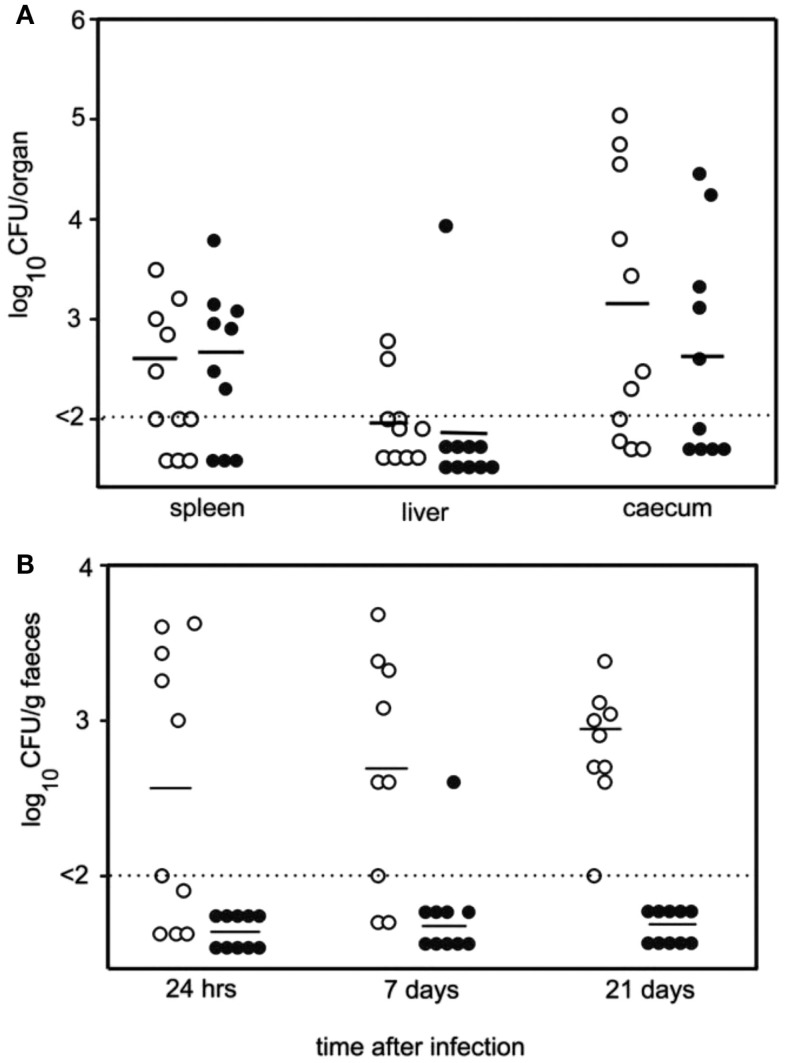
**TLP is required for survival of *Salmonella typhimurium* in high uric acid environments such as chicken feces**. **(A)**. Numbers of viable bacteria in the spleen, liver and cecum of hens 7 days after oral inoculation with either wildtype *Salmonella* (open circles) or TLP null *Salmonella* (closed circles). Horizontal lines: average number of bacteria per organ; dotted line: lower detection limit of experiment. **(B)**. Numbers of viable bacteria in the feces of hens up to 21 days following oral inoculation with either wildtype *Salmonella* (open circles) or TLP null *Salmonella* (closed circles). Horizontal lines: average number of bacteria per gram feces; dotted line: lower detection limit of experiment. There was a significantly lower number of TLP null *Salmonella* than wildtype *Salmonella* in the feces and each time point measured (30).

Hens were inoculated with either the wildtype or the TLP knock-out strain of *Salmonella*. The feces of hens inoculated with the TLP knock-out strain of *Salmonella* contained significantly less live *Salmonella* than the feces of the hens inoculated with the wildtype *Salmonella* (Figure 9) (30). This demonstrated that TLP was important for the survival of *Salmonella* in high uric acid environments. Thus, not only is it important to choose the most appropriate animal model for such experiments, but this clearly showed that TLP is important in the survival of *Salmonella* in high uric acid environments.

## Mice Lacking TLP Suffer Toxicity from Uric Acid Oxidation Intermediates

Mice lacking TLP were generated and found to have increased thrombopoietin synthesis by the liver and enlarged livers (hepatomegaly) resulting in increased platelet counts in the blood (thrombocytosis). Most mice lacking TLP also developed hepatocellular carcinoma (42). It was concluded that this phenotype was due to the toxic uric acid oxidation intermediates resulting from the lack of 5-HIUase in the cytoplasm of the mouse hepatocytes.

## Conclusion

TLP/TTR is an excellent model for the study of protein evolution. Notably because (i) it is found in all kingdoms, (ii) it has a stable structure, (iii) it lacks post-translational modification, (iv) it can be directed to many sites within a cell or secreted, (v) modification of just a few amino acids in the active site changed its function from a 5-HIUase to a T3 distributor, (vi) successive shifts in the position of the intron 1–exon 2 splice site changed TTR from a T3 distributor to a T4 distributor. Thus, apparently two different molecular mechanisms have resulted in changes in the functions of TLP/TTR. The functions of TLPs in plants and other organisms are likely to be different to those in *Salmonella* or mice. The differing signal peptides for the various groups of TLPs could be interrogated in conjunction with RNAseq analyses to gain insights into the functions of TLPs in various organisms. The suggestion that cytoplasmic and periplasmic TLPs within a species evolved in separate pathways suggests divergence of functions between these groups of compartmentally distinct TLPs. Presumably, additional evolutionary mechanisms have also been used to modify the functions of TLPs in each compartment in these species. These mechanisms remain to be investigated.

## Conflict of Interest Statement

The author declares that the research was conducted in the absence of any commercial or financial relationships that could be construed as a potential conflict of interest.

## Abbreviations

TTR transthyretin, TLP transthyretin-like protein, 5-HIUase 5-hydroxyisourate hydrolase.
